# Wideband Doherty Power Amplifier: A Design Approach

**DOI:** 10.3390/mi13040497

**Published:** 2022-03-23

**Authors:** Jorge Julián Moreno Rubio, Edison Ferney Angarita Malaver, Luis Ángel Lara González

**Affiliations:** 1 Grupo de Investigación en Telecomunicaciones—GINTEL, Universidad Pedagógica y Tecnológica de Colombia, Sogamoso 152211, Colombia; edison.angarita@uptc.edu.co; 2 Instituto de Recursos Minero-Energéticos—IRME, Universidad Pedagógica y Tecnológica de Colombia, Sogamoso 152211, Colombia; luisangel.lara@uptc.edu.co

**Keywords:** GaN-based FETs, wideband Doherty power amplifier, broadband matching networks

## Abstract

This paper presents a simple method to design wideband Doherty power amplifiers (DPAs) based on the synthesis of a combiner network which can mimic the response of an ideal compensation of the device reactive output equivalent network and exploit the maximum power capabilities of the device. Using the Wolfspeed’s CGH40006 and CG2H40025 GaN HEMT devices, two DPAs were designed and simulated to demonstrate the effectiveness of the proposed approach. In both cases, a 1.4 GHz bandwidth was obtained together with an efficiency higher than 44 and 49% at 6 dB OBO. The saturated output power was higher than 41.2 and 47 dBm over the band, for the DPAs using the CGH40006 and CG2H40025 devices, respectively.

## 1. Introduction

The power amplifier (PA) is a key element of a wireless communication system transmitter. This is the circuit which transforms the energy coming from DC sources into RF energy, turning it into the main consumer of energy in the system. On the other hand, the need to increase the spectrum utilization and data transmission rate in 4G and 5G wireless networks has led to complex modulation schemes, which then creates modulated signals with a high peak-to-average power ratio (PAPR) [1,2]. This characteristic of the system demands high energy efficiency in both power back-off and full saturation. Additionally, broadband requirements are common features of modern communication systems. As such, each circuit must be designed with this characteristic [3].

By virtue of these facts, outphasing [4], Doherty, and load-modulated [5,6] PAs have been proposed in the literature. In this context, the Doherty power amplifier (DPA) is considered as one of the most useful and well-established solutions. This is not only because of its good efficiency in a large power regime, but also due to its simplicity in comparison with other architectures that mostly involve the additional digital control processing of signals, which makes them much more complicated.

The importance of the DPA design for broadband applications is evidenced by many publications which have explored solutions to extend the bandwidth. For example, in [7], a broadband DPA was designed using a technique supported by an initial estimation of the bandwidth which required nothing more than linear simulations. A nonterminated branch line coupler was used as an output combiner in [8] to improve the bandwidth. Wu et al. improved the bandwidth of a DPA through a Klopfenstein taper in [9], while in [10], a three-port input and output network technique is proposed to overcome the bandwidth constraints of the DPA. Despite these proposals, a direct and general solution to obtain wideband DPAs is not clearly supported for different sizes of transistors.

In this paper, a simple but effective methodology to design wideband DPAs is introduced. It is based on the design of closed equations which immediately lead to a wideband combiner network. The effectiveness of the proposed methodology is verified through the design of two different DPAs using devices with different power capability. The first DPA was designed employing a 6 W GaN HEMT device, while the second one used a 25 W GaN HEMT. In both cases, competitive results, in terms of efficiency, output power, and bandwidth, were obtained [7,8,11,12], demonstrating a high level of generalization for the proposed methodology.

## 2. Estimation of the Device’s Output Reactive Network

**Figure 1:**
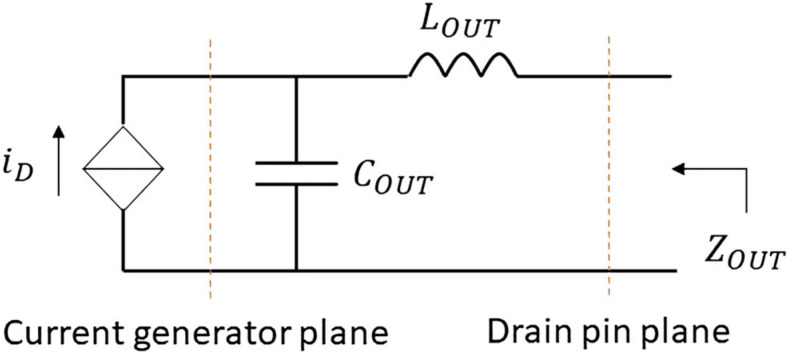
Equivalent output reactive network.

Let us consider the network shown in Figure 1, in which a current generator is in parallel with a capacitor $C_{\mathrm{OUT}}$, while an inductor $L_{\mathrm{OUT}}$ is in series to them. The current generator represents the drain current of a field effect transistor (FET), and $C_{\mathrm{OUT}}$ and $L_{\mathrm{OUT}}$ stand for its equivalent output reactive network, as proposed in [13,14,15,16,17]. From this perspective, the current generator can be turned off by just applying a gate voltage $V_{G}$ lower than the device’s threshold voltage $V_{\mathrm{thr}}$. Under this condition, $C_{\mathrm{OUT}}$ and $L_{\mathrm{OUT}}$ can be estimated by looking at the output impedance $Z_{\mathrm{OUT}}$ (see Figure 1) at two (or more) different frequencies.

If the drain current is zeroed (i.e.,  $i_{D}=0$) in Figure 1, it means that the current generator is equivalent to an open circuit. Meanwhile, the impedance $Z_{\mathrm{OUT}}$ will be given by

(1) $$Z_{\mathrm{OUT}}=j\left({\omega L}_{\mathrm{OUT}}-\frac{f}{{\omega C}_{\mathrm{OUT}}}\right)$$

where f is the frequency of interest and ω=2πf. Thus, $Z_{\mathrm{OUT}}$ can be simulated using the nonlinear model of the device, under the condition of a gate voltage $V_{G}<V_{\mathrm{thr}}$. Then, using Equation (1), $L_{\mathrm{OUT}}$ and $C_{\mathrm{OUT}}$ are estimated.

Since the purpose of this paper is to present a methodology as analytical as possible through which the designer will have full command of the design process, the optimal load is defined theoretically rather than experimentally (e.g., load pull). Thus, as reported in [18], considering tuned load and class B conditions, the optimal load to be synthesized at the current generator reference plane (see Figure 1) is obtained by

(2) $$Z_{\mathrm{opt}}=\frac{2\left(V_{\mathrm{DD}}-V_{k}\right)}{I_{\mathrm{MAX}}}$$

where $V_{\mathrm{DD}}$ is the drain bias voltage, $V_{k}$ is the knee voltage, and $I_{\mathrm{MAX}}$ is the maximum current, as shown in [18].

Since the capacitance $C_{\mathrm{OUT}}$ represents a relatively small magnitude impedance at the design frequencies, for simplicity, the second, third, and higher harmonic loads will be considered within a short circuit. This consideration permits the use of Equation (2) to calculate the optimal load. However, the second harmonic, at the very least, should be monitored during the design process. Fortunately, many simulation tools are available nowadays to do so. Moreover, the optimal load for class B conditions is always close to the optimal load for class AB, and it is simpler to calculate.

## 3. Ideal Compensation of $C_{OUT}$ and $L_{OUT}$

**Figure 2:**
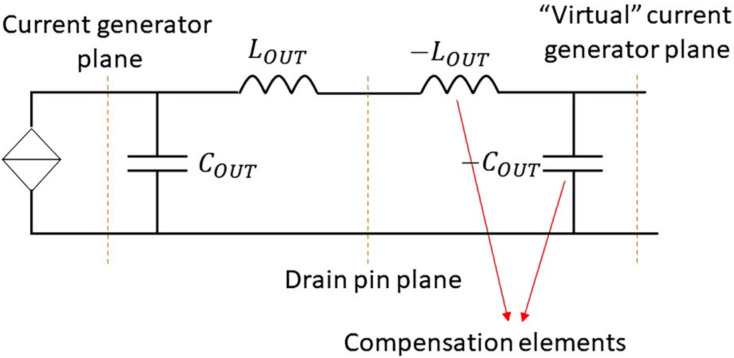
Ideal externalization of the current generator plane.

Starting from the estimation of the device output equivalent network and assuming a symmetric DPA (the same device for the main and auxiliary amplifiers), an usual next step is the compensation of $C_{\mathrm{OUT}}$ and $L_{\mathrm{OUT}}$ to eliminate the problem of a frequency dependent load, which is one of the main bandwidth limitation issues. If ideality is allowed for the sake of better understanding, the obvious and perfect solution is the inclusion of a negative series inductance and a negative shunt capacitor, connected to the drain pin of the device, as sketched in Figure 2. Consequently, it can be said that the intrinsic device’s drain has been virtually accessed. In other words, the current generator reference plane is externalized.

**Figure 3:**
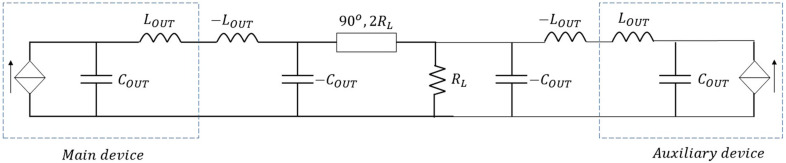
DPA output network with ideal compensation of $C_{\mathrm{OUT}}$ and $L_{\mathrm{OUT}}$.

Within this context, an output network for a DPA can be designed, as explained in [18]. Figure 3 shows the DPA output network with an ideal compensation of the reactive elements $C_{\mathrm{OUT}}$ and $L_{\mathrm{OUT}}$ which uses a π/2 electrical length transmission line as impedance inverter. Notice that, in this case, the bandwidth limitation is only governed by the impedance inverter, at least at the output. Assuming a 6 dB Doherty region, $R_{L}=Z_{\mathrm{opt}}/2$.

To evaluate the bandwidth of this ideal approach, two DPAs are designed using the Wolfspeed CGH40006 and CG2H40025 GaN devices. For these devices, the optimal load is estimated as $Z_{\mathrm{opt}}=42\;\mathrm{and}\;14\;Ω$ ($R_{L}=21\;\mathrm{and}\;7\;Ω$), whereas from Equation (1), the reactive components $C_{\mathrm{OUT}}=1.19\;\mathrm{and}\;3.39\;\mathrm{pF}$ and $L_{\mathrm{OUT}}=0.46\;\mathrm{and}\;0.49\;\mathrm{nH}$ are obtained, respectively. As an example in this regard, the impedance inverter is designed at a reference frequency f=3 GHz.

**Figure 4:**
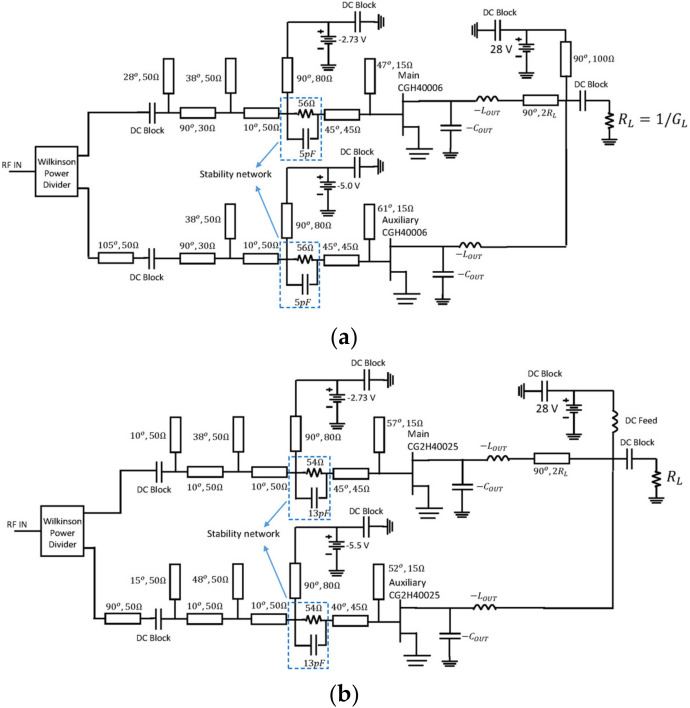
DPA schematic with ideal compensation of $C_{\mathrm{OUT}}$ and $L_{\mathrm{OUT}}$: using the (**a**) CGH40006 and (**b**) CG2H40025.

**Figure 5:**
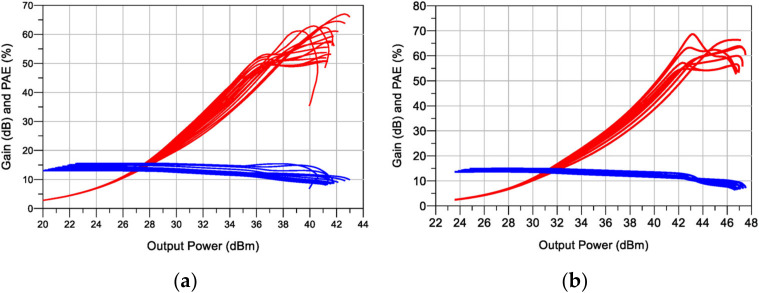
Simulated Gain and PAE versus output power of the “idealized compensated” DPA: 2 to 3.4 GHz: using the (**a**) CGH40006 and (**b**) CG2H40025.

The schematics of the designed DPAs are shown in Figure 4. Notice that the input matching networks (IMNs) for the main and auxiliary devices are designed, including a stability network and a bias tee which is implemented utilizing a high impedance (80 Ω) transmission line with π/2 of electrical length at f=3 GHz. The effect of adding the bias-Tee over the DPA performance is not significant, and therefore, no adjustment to the other elements is necessary. Figure 5 shows the efficiency and gain profiles versus output power of the designed DPAs for a frequency sweep from 2 GHz to 3.4 GHz. These simulations demonstrated a 1.4 GHz potential bandwidth between 2 and 3.4 GHz, under the condition of compensating perfectly $C_{\mathrm{OUT}}$ and $L_{\mathrm{OUT}}$.

## 4. Proposed Solution

**Figure 6:**
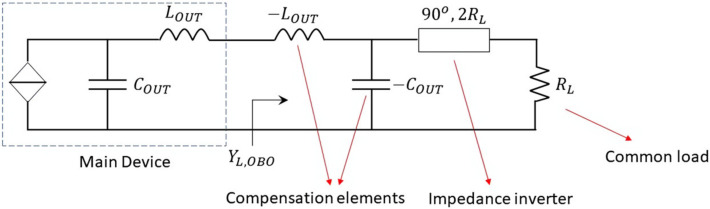
Main amplifier ideal output network in back-off.

By observing the solution shown in Figure 4, the main amplifier output network can be simplified at back-off, as depicted in Figure 6. Notice that, as usual, the term “back-off” refers to the turned-off condition of the auxiliary amplifier. In this way, the admittance seen by the main device, at the frequency of interest, can be calculated as

(3) $$Y_{L,\mathrm{OBO}}=Y_{1}={\left(-{j\omega L}_{\mathrm{OUT}}-\frac{4R_{L}\left(\frac{1}{{j\omega C}_{\mathrm{OUT}}}\right)}{4R_{L}-\frac{1}{{j\omega C}_{\mathrm{OUT}}}}\right)}^{-1}=P_{1}+{\mathrm{jQ}}_{1}$$

Thus, $Y_{1}$ is the ideal admittance at the external drain pin plane, at 6 dB power back-off, as a function of frequency.

**Figure 7:**
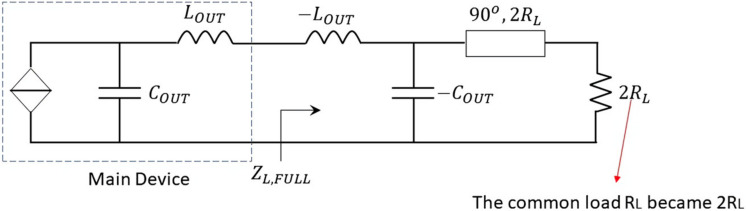
Main amplifier “ideal” output network in “full-power”.

The full-power condition of the DPA means that both main and auxiliary devices are current and voltage saturated. The equivalent output circuit for the main amplifier is presented in Figure 7. The common load $R_{L}$ becomes $2R_{L}$, owing to the load modulation produced by the auxiliary amplifier current. In this case, the admittance at the drain pin is given by

(4) $$Y_{L,\mathrm{FULL}}=Y_{2}={\left(-{j\omega L}_{\mathrm{OUT}}-\frac{2R_{L}\left(\frac{1}{{j\omega C}_{\mathrm{OUT}}}\right)}{2R_{L}-\frac{1}{{j\omega C}_{\mathrm{OUT}}}}\right)}^{-1}=P_{2}+{\mathrm{jQ}}_{2}$$

**Figure 8:**
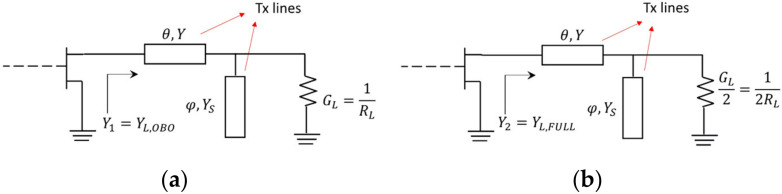
Main amplifier output network in (**a**) back-off and (**b**) full-power.

$Y_{1}$ and $Y_{2}$ are the initial point of the proposed methodology. Using these two admittance values, a synthesis of a quite simple but real OMN is carried out. This network emulates the effects of the negative inductor ($-L_{\mathrm{OUT}}$), the negative capacitor ($-C_{\mathrm{OUT}}$), and the impedance inverter in Figure 4. For this purpose, a series transmission line connected to an open stub is proposed. The solution for back-off and full-power conditions are shown in Figure 8a,b, respectively.

Thus, from Figure 8a, the following relation is obtained:

(5) $$Y_{1}=P_{1}+{\mathrm{jQ}}_{1}=Y\frac{G_{L}+j\left(B+\mathrm{YT}\right)}{\left(Y-\mathrm{BT}\right)+{\mathrm{jG}}_{L}T}$$

where T=tanθ, $B=Y_{S}\tan\varphi$, while $Y_{1}$ is calculated using Equation (3)

(6) $$\left\{\begin{array}{c} P_{1}\left(Y-\mathrm{BT}\right)-Q_{1}G_{L}T={\mathrm{YG}}_{L}\\ Q_{1}\left(Y-\mathrm{BT}\right)+P_{1}G_{L}T=Y\left(B+\mathrm{YT}\right) \end{array}\right.$$

Similarly, from Figure 8b

(7) $$Y_{2}=P_{2}+{\mathrm{jQ}}_{2}=Y\frac{\frac{G_{L}}{2}+j\left(B+\mathrm{YT}\right)}{\left(Y-\mathrm{BT}\right)+j\frac{G_{L}}{2}T}$$

with $Y_{2}$ obtained from Equation (4), or

(8) $$\left\{\begin{array}{c} P_{2}\left(Y-\mathrm{BT}\right)-Q_{2}\frac{G_{L}}{2}T=Y\frac{G_{L}}{2}\\ Q_{2}\left(Y-\mathrm{BT}\right)+P_{2}\frac{G_{L}}{2}T=Y\left(B+\mathrm{YT}\right) \end{array}\right.$$

By solving Equations (6) and (8) simultaneously for B, Y**,** and T, the following design closed equations are obtained

(9) $$\left\{\begin{array}{c} B=\frac{Q_{2}-Q_{1}+\left(\frac{P_{2}}{2}-P_{1}\right){\mathrm{MG}}_{L}}{\left(Q_{2}-Q_{1}\right)M}\\ Y=\sqrt{\frac{Q_{1}\left(1-\mathrm{BM}\right)+P_{1}G_{L}M-B}{M}}=\sqrt{\frac{Q_{2}\left(1-\mathrm{BM}\right)+P_{2}\frac{G_{L}}{2}M-B}{M}}\\ T=MY \end{array}\right.$$

where $M=\left(P_{1}-2P_{2}\right)/\left(2P_{2}Q_{1}-P_{1}Q_{2}\right)$.

**Figure 9:**
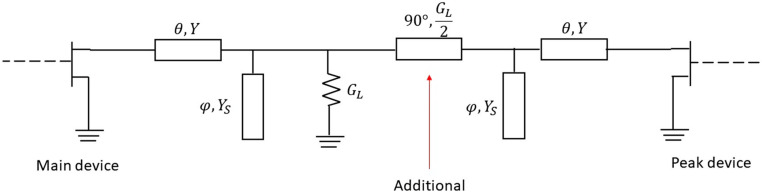
Proposed combiner topology: wideband solution.

Finally, the output network for the auxiliary device is proposed as a replica of the one for the main device while adding a 90° transmission line before the common load, as shown in Figure 9, with $2R_{L}$ as characteristic impedance. From T, θ can be obtained as θ=arctanT, while $Y_{S}$ and φ can be extracted from $\varphi =\arctan\left(B/Y_{S}\right)$. In the latter case, $Y_{S}$ can be assumed as a free selection variable, and then φ is obtained.

Using Equation (9) with a reference frequency of 3 GHz, the obtained calculations for the CGH40006 and CG2H40025 Wolfspeed devices are shown in Table 1.

**Figure 10:**
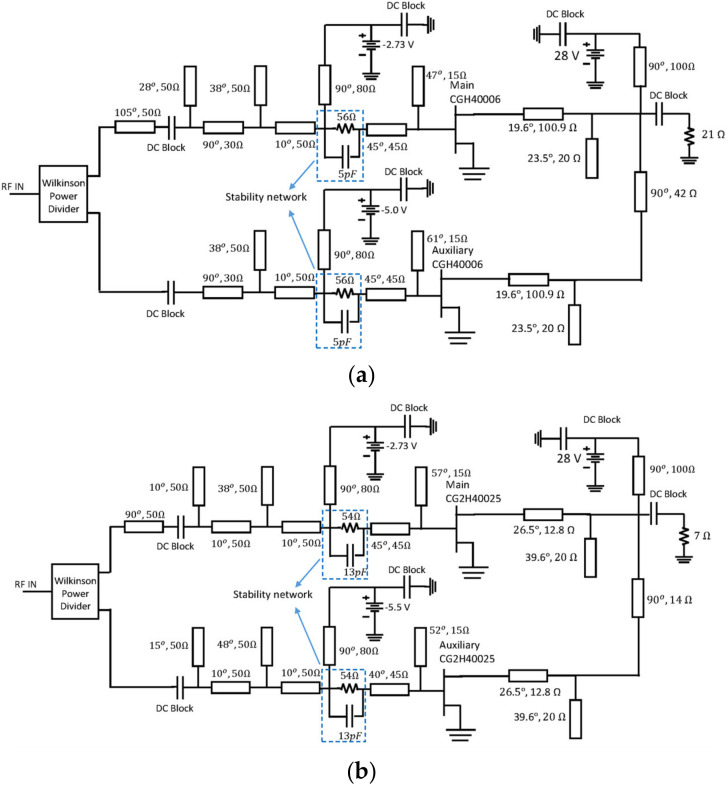
Designed DPAs: (**a**) using the CGH40006 and (**b**) CG2H40025.

**Figure 11:**
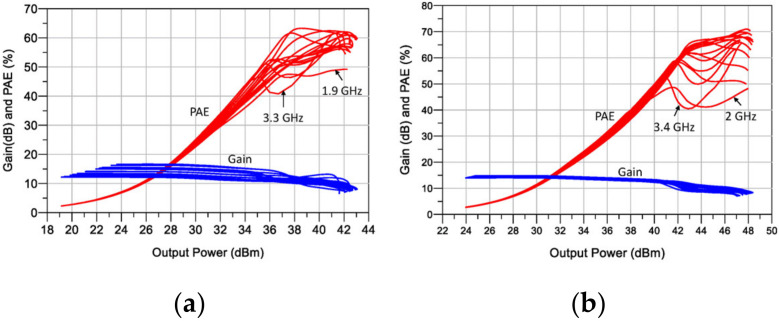
Simulated gain and PAE versus output power of the proposed DPAs: (**a**) using the CGH40006 and (**b**) CG2H40025.

**Figure 12:**
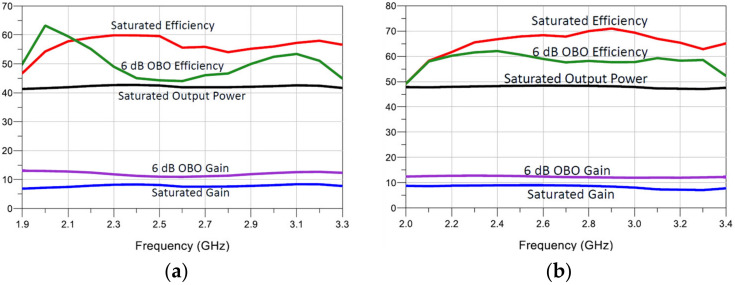
Simulated gain, PAE, and output power versus frequency of the proposed DPAs: (**a**) using the CGH40006 and (**b**) CG2H40025.

The schematics of the designed DPAs are presented in Figure 10. The simulated PAE and gain profiles are shown in Figure 11, while Figure 12 presents the gain, output power, and PAE versus frequency. As can be noticed, the obtained bandwidth is comparable with the ideal compensation one (see Figure 5). Moreover, in this case, a bandwidth of 1.4 GHz was obtained in both circuits.

For the first DPA, a maximum output power between 41.2 and 42.6 dBm was obtained over the band, together with a 6-dB-OBO PAE from 44% to 63%. The saturated PAE was in the range of 47% to 60%. The second DPA obtained a maximum power between 47 and 48.4 dBm and a 6-dB-OBO PAE from 49% to 62%, with full-power PAE from 49% to 71%. The presented results show a full exploitation of the devices’ power capabilities.

## 5. Conclusions

This paper presents a simple methodology to design wideband DPAs. This exploits a combiner network implemented using a series transmission line connected to an open stub one, which imitates the conditions obtained using a negative capacitor and inductor to compensate the intrinsic reactive device’s output network. The combiner can be easily calculated using closed equations. As a case study, two DPAs are designed using 6 W and 25 W GaN HEMT devices. The obtained simulation results show the potential of the presented approach.

## Figures and Tables

**Table 1:** Calculations for the CGH40006 and CG2H40025 GaN HEMT devices.

Device	Ref. Freq.	$L_{OUT}$	$C_{OUT}$	$R_{L}$	θ	1/Y	φ	$1/Y_{S}$
CGH40006	3 GHz	0.46 nH	1.19 pF	21 Ω	19.6°	100.9 Ω	23.5°	20 Ω
CG2H40025	3 GHz	0.49 nH	3.39 pF	7 Ω	26.5°	12.8 Ω	39.6°	20 Ω
